# Review of surgical treatment of iatrogenic iliofemoral artery injury in the pediatric population after catheterization

**DOI:** 10.1186/s40001-023-01510-y

**Published:** 2023-11-16

**Authors:** Rodrigo Lozano-Corona, Adriana Torres-Machorro, Reinhard Ortiz-Beitz, Aristeo Reyes-Monroy, Ignacio García-Lugo, Christopher Ruben-Castillo, Luis Angel Guerrero-Galindo

**Affiliations:** 1 Department of Surgery, Section of Vascular Surgery and Endovascular Therapy, Hospital Regional Licenciado Adolfo Lopez Mateos (Instituto de Seguridad y Servicios de Salud de los Trabajadores del Estado), Unuversidad Av 1321, Zip Code 01030, Mexico City, Mexico; 2 Department of Surgery, Section of Vascular Surgery and Endovascular Therapy, Instituto Nacional de Cardiología “Ignacio Chávez”, Mexico City, Mexico; 3 Department of Surgery, Medica Sur, Mexico City, Mexico; 4 Department of Surgery, Section of Vascular Surgery and Endovascular Therapy, Instituto Nacional de Ciencias Médicas y Nutrición “Salvador Zubirán”, Mexico City, Mexico

**Keywords:** Pediatrics, Vascular trauma, Iliofemoral arteries, Iatrogenic injury

## Abstract

Trauma is the leading cause of death in the pediatric population. Although vascular trauma has an incidence of 6% in civilian population, iatrogenic injuries are the leading cause, and the most frequent injured vessel is the iliofemoral sector. However, little information is available and there are no guidelines about its treatment. Therefore, this review aimed to describe the information available concerning pediatric iatrogenic arterial trauma, focusing on the iliofemoral segment and present 3 cases. We described 11 articles with 171 patients, of whom 61% underwent surgery to treat iatrogenic trauma. Mean age was 3.28 years (standard deviation of 3.5 years), and 54% were female. Most iliofemoral injuries occurred after arterial catheterization for hemodynamic monitorization and therapeutic or diagnostic cardiac catheterization (due to congenital heart diseases, including septal defects, tetralogy of Fallot, aortic coarctation, and patent ductus arteriosus). For acute complications, arterial thrombosis was the leading injury, followed by pseudoaneurysm, hematoma, dissection, transection, avulsion, eversion, and combined lesions.

## Introduction

Pediatric trauma is the leading cause of death in children above 1 year in the United States of America. Vascular trauma is rare, but a potential cause of death, and reports account for 0.6–1% of the pediatric population [1]. Remarkably, the most frequent cause of pediatric vascular trauma is iatrogenic injury (50%), which occurs mainly after interventions in peripheral vessels during catheters placing, arterial punctures for blood gas analysis, hemodynamic monitorization, endovascular diagnostic procedures, umbilical artery catheterization or during open surgery near a vascular territory. Iatrogenic trauma has different injury, morbidity, and outcome patterns than war or civilian vascular trauma [2].

The iliofemoral axis is the most common access site for diagnostic and therapeutic catheterization procedures; in addition, when catheterizations are therapeutic, larger caliber of endovascular devices is often used. Therefore, the most common complications of these procedures occur in the site of access (iliofemoral arteries), including hematoma, occlusion, bleeding, arterial dissection, arteriovenous fistula, pseudoaneurysm formation or chronic complications, such as pain. The risk of developing these complications has been estimated from 2 to 40% among pediatric patients [3]. In addition, pediatric vascular trauma is more difficult to surgically treat from a technical perspective due to the small diameter of the injured vessel, the spasticity of young arteries and the inherent difficulties of choosing a treatment that should be adapted to the continuous axial growth. Limited published reports and the lack of reference guidelines which homogenize management of iatrogenic vascular trauma in pediatric population make treatment choices more complex [4].

Therefore, this study aims to describe the information reported in the scientific literature concerning surgical scenarios of pediatric iatrogenic trauma focusing on the iliofemoral arteries and presenting three clinical cases.

## Methods

To introduce the literature review, we first described three clinical cases of pediatric iatrogenic vascular trauma in two national reference centers in Mexico. Data were retrieved from electronic medical records and surgical notes.

Then, a literature review was performed among PUBMED and EBSCO databases using the words “pediatric iliac artery injury” or “pediatric iatrogenic iliac artery trauma OR injury”. Inclusion criteria were papers written in English, German, and Spanish from 1998 to January 25th, 2023. Exclusion criteria were civilian trauma, venous trauma, iatrogenic injury in other arteries, no specifications about treatment, medical treatment only and reports that included data from adult patients. In addition, the patient’s demographics, the underlying pathology that justified the treatment in which iatrogenic trauma occurred, the injured artery, the treatment granted, and the post-treatment follow-up of the arterial lesion were retrieved from each report.

Iatrogenic vascular trauma was divided into acute (those who received treatment within 30 days of injury) and chronic (those who received treatment after 30 days of injury). Demographic data are shown using descriptive statistics.

## Results

### Case 1

**Fig. 1:**
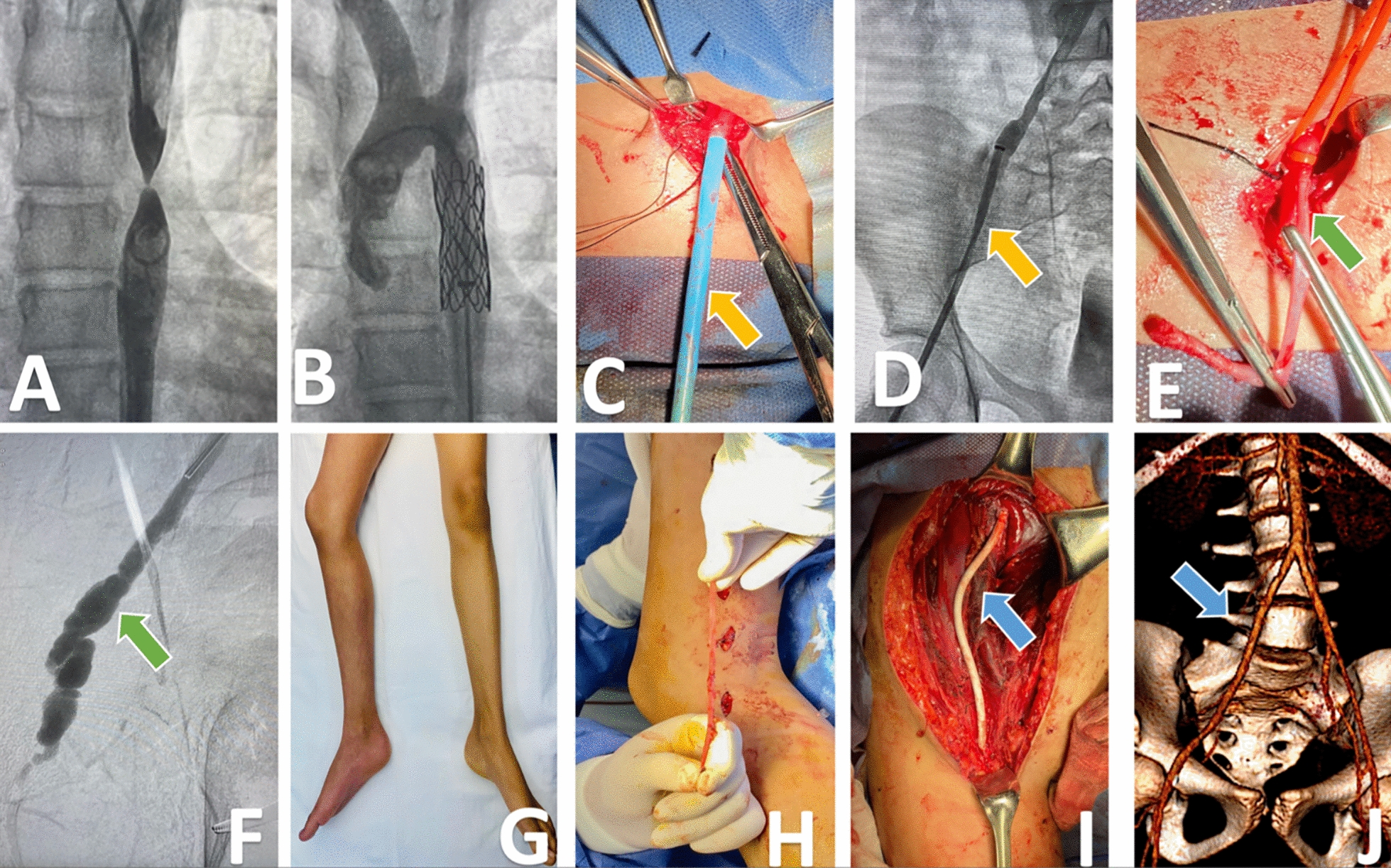
From left to right and chronologically, the images of the case of a 12-year-old female patient who underwent aortic thoracic stent placement due to aortic coarctation (**a** and **b**), with the impossibility to remove the 12 Fr sheath once the procedure was completed. **c** Yellow arrow signaling the sheath inside the common femoral artery, **d** the tip of the sheath in the common iliac artery, yellow arrow signaling a point of narrowing in the external iliac artery (EIA), **e** green arrow indicates eversion of the intima and media layers of the external iliac artery through the femoral puncture once the introducer was removed, **f** green arrow indicates an arteriography image of EIA pseudoaneurysm with imminent rupture risk after sheath removal. **g** Right lower limb with cyanosis. **h** Great saphenous vein harvested and unsuitable as a graft. **i** Blue arrow indicates the interposition of ePTFE 6 mm graft from the internal iliac artery to the common femoral artery as a method of vascular reconstruction, **j** 3D reconstruction of postoperative abdominopelvic Angio CT, where the patency of the PTFE graft is evident and signaled with a blue arrow

A 12-year-old female patient with a history of newly diagnosed aortic coarctation (AoCo) underwent a percutaneous aortic catheterization through the right common femoral artery (CFA) with a 6 Fr sheath that was later changed to a 12 Fr long sheath (55 cm) for coarctation site dilatation and 12 mm-diameter stent deployment. Post-procedural arteriographic control was satisfactory at the aorta level; however, when attempting the remotion of endovascular devices, the 12 Fr sheath remained fixed and could not be easily removed. A slight eversion of the endothelium was noticed after persistent traction. Hence, after emergent vascular surgery consultation, a femoral incision, under fluoroscopic guidance, was made to obtain vascular control and radial access to retrieve the sheath. The 12 Fr sheath was removed after traction, but unfortunately, the tunica intima and the tunica media of the external iliac artery (EIA) were everted, conditioning a common femoral artery (CFA) occlusion. The post-sheath retrieval arteriography demonstrated an EIA pseudoaneurysm contained only by the tunica adventitia. Therefore, an open surgical repair was performed through a right hockey-stick incision. Simultaneously, a great saphenous vein (GSV) was harvested but was unsuitable as a graft due to its small diameter. The EIA was identified and repaired with a 6 mm ePTFE graft interposition. Graft choice was selected based on native measurements of the superficial femoral artery (5.0–5.5 mm) and the common iliac artery (CIA) (6.0–6.5 mm). Anastomosis between the CIA and the distal third of the CFA was performed with interrupted suture repair with 6–0 polypropylene. The patient had favorable evolution, and the 6-month follow-up ankle–brachial index remained normal (0.9) in the intervened lower limb, with palpable distal pulses and no signs of claudication (Fig. 1).

### Case 2

**Fig. 2:**
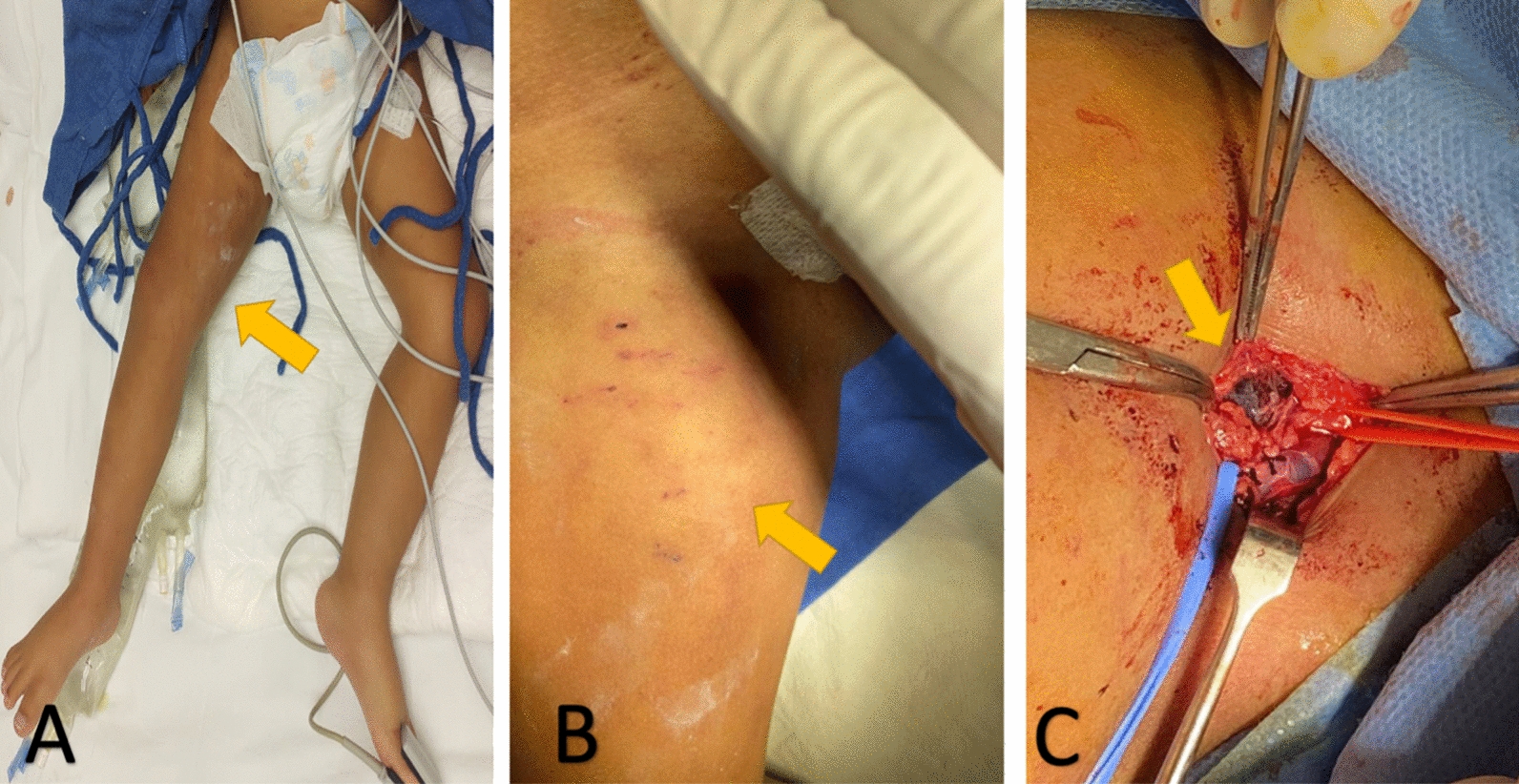
Images of the case of a 2-year-old male patient who underwent right femoral artery catheterization to hemodynamic monitoring and developed ALLI of the right lower limb. **a** Yellow arrow signaling cyanosis of the right lower limb, **b** yellow arrow signaling groin hematoma, **c** yellow arrow indicates hematoma over the CFA

A 2-year-old male diagnosed with trisomy 21 underwent UCI hospitalization secondary to septic shock for pneumonia. After a right femoral artery catheterization for hemodynamic monitoring, he developed acute lower limb ischemia (ALLI), so the catheter was removed, and systemic heparin started, but ALLI symptoms persisted. The patient underwent surgical treatment; CFA thrombectomy was performed with 2 Fr Fogarty catheter and arterial interrupted suture repair with 7/0 polypropylene suture. After 1 year of follow-up, no signs of recurrent ischemia were found, and normal growth chart was as expected (Fig. 2).

### Case 3

**Fig. 3:**
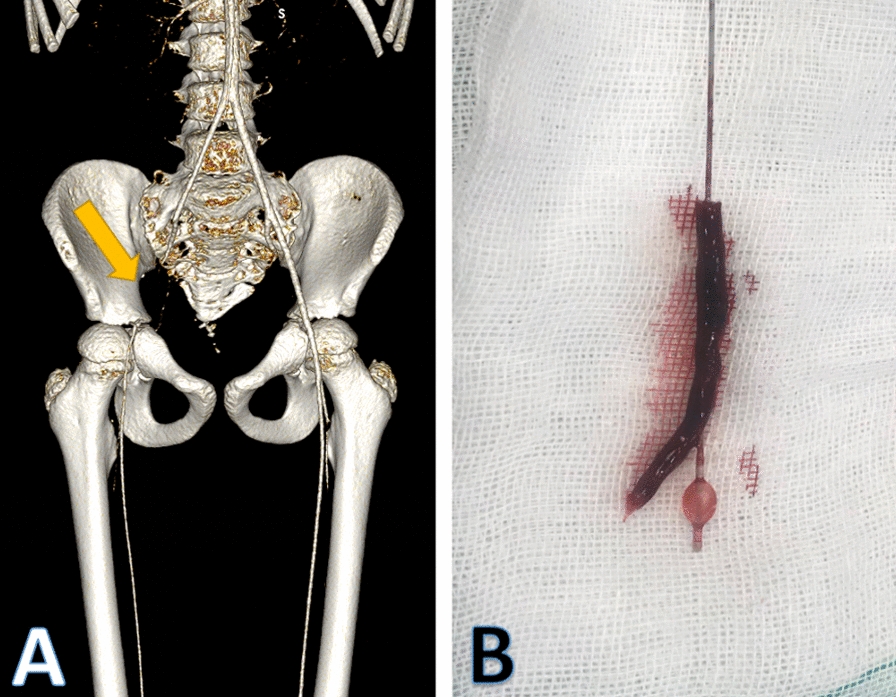
Images of the case of a 9-year-old male patient who underwent right femoral artery diagnostic catheterization before surgical repair of interventricular communication. **a**: Angio CT of iliac and femoral arteries, chondral spaces are appreciated in iliac bones, yellow arrow signaling right EIA thrombosis, **b** right EIA thrombus removed into the 2Fr Fogarty catheter

A 9-year-old male with the antecedent of pulmonary atresia and interventricular communication underwent hospitalization for septum defect treatment. A diagnostic catheterization was performed through the right CFA before surgical closure of interventricular communication. The patient developed ALLI of the right lower limb 24 h after cardiac surgery, and EIA thrombosis was documented by angioCT. A right CFA thrombectomy was performed with 2 Fr Fogarty catheter, and arterial interrupted suture repair with 7/0 polypropylene suture. After 1 year of follow-up, he showed no signs of recurrent ischemia, a normal growth chart as expected, and no limitations in his physical activities (Fig. 3).

### Literature review

We selected 64 articles, of which 52 were excluded, because they were related to civil or non-iatrogenic-specified trauma, involved other arterial territories (intracranial or upper extremities), or included adult cases. Finally, 12 articles were included, comprising 171 patients of whom 105 (61%) underwent surgical treatment of iatrogenic trauma (including our 3 cases).

The mean age was 39.4 months or 3.28 years (SD 42.9 months or 3.5 years), and 48% (n = 82) of the injuries occurred in female patients. Iliofemoral injuries occurred after arterial catheterization as follows: 52 for hemodynamic monitorization, 75 for therapeutic or diagnostic cardiac catheterization due to congenital heart disease, including septal defects (*n* = 8), tetralogy of Fallot (*n* = 6), aortic coarctation (*n* = 6), patent ductus arteriosus (*n* = 4). Seven patients underwent ECMO, and the rest was not well-specified. The most affected arteries were the CFA (*n* = 67, 73%), followed by EIA (*n* = 13, 15%), the CIA (*n* = 1), and not specified in the rest of cases (referred only as iliofemoral segment).

Among the acute complications (*n* = 144), mean age was 3.1 years (SD 3.4), 50% occurred in female patients, arterial thrombosis was the leading injury (*n* = 109, 35 underwent surgical treatment) followed by pseudoaneurysm (*n* = 7), hematoma (*n* = 5), AVF (*n* = 5), dissection (*n* = 4), transection (*n* = 2), avulsion (*n* = 2) combined lesion (*n* = 3), eversion (*n* = 1) or others (in 6 cases this information was not specified). Table 1 resumes the type of surgical repair according to each injury; most required thrombectomy and arterial closure by an interrupted suture repair, Saphenous vein patch angioplasty (SVPA) of Great Saphenous Vein (GSV), femoral vein or PTFE graft interposition. Bypass was performed in 4 cases of iliofemoral dissection, iatrogenic injury during surgery, amplatzer occluder migration to CIA, and eversion of EIA. The post-treatment results were satisfactory in 91% of patients, with follow-ups ranging from 0 to 118 months (an average of 135 months). Outcomes were reported as pulses regained and no signs or symptoms of ischemia. However, mortality was reported in 3 cases of < 2-year-old patients, bilateral lower limb amputation in one case, affected lower limb amputation in one case, one reoperation due to bypass occlusion, one bypass stenosis, one case of deep vein thrombosis and 3 cases of wound complications.

**Table 1:** Patient’s characteristics

Author years	Type of study/Number of patients	Mean age/Gender/underlying condition	Anatomical site of injury/trauma mechanism/type of injury	Type of repair	Results and follow-up
Pigula [5] (2000)	Case report/1	6 mo M/type B interrupted aortic arch	R EIA/TCC/acute bleeding/hematoma secondary to artery transection	Retroperitoneal approach and AISR	Follow-up: 4 mo BPG of 10 mmHg
Lin [2] (2001)	Retrospective cohort/27 underwent surgery for acute complications	4.8 y (Range, 1 w to 17.4 y)/ M = 19, F = 15/NS	CFA/DCC (70.2%) or TCC (58.8%) - ALLI, thrombosis (*n* = 14) - PsA (*n* = 4) - AVF (*n* = 5) - Bleeding/hematoma (*n* = 4)	-ALLI (*n* = 14): Thrombectomy + AISR (*n* = 6), or thrombectomy + resection with E-E anastomosis (*n* = 2), or thrombectomy plus SVPA (*n* = 6) - PsA: AISR - FAV: AISR, vein ligation or suture repair - Bleeding/hematoma: AISR	Mean follow-up: 38 mo (range, 8 to 62) No limb loss, 85% regain normal circulation 30-day mortality of 3% (*n* = 3) 12% overall morbidity
Dogan [6] (2006)	Retrospective cohort/2	2 y NS/NS	Right CFA/ALLI, embolus (*n* = 1)/ Right CFA pseudoaneurysm (*n* = 1)/ Both after failed venipuncture	Embolectomy (*n* = 1) End-to-end anastomosis of CFA with 9/0 polypropylene (*n *= 1)	NS
Aspalter [7] (2007)	Retrospective cohort/8	5.8 y (0.3 to 10.9 y)/F = 2, M = 4/ Ao Co (*n* = 2) ARDS (*n* = 1) SeD (*n* = 1) TOF (*n* = 1) NoCC (*n* = 3)	CFA: - DCC (*n* = 1) - TCC (*n* = 4) - Failed venipuncture (*n* = 3) - ALLI, thrombosis (*n* = 3) - PsA (*n* = 1) Psa + ALLI (*n* = 1) PsA + AVF (*n* = 1) PsA + bleeding (*n* = 1) CFA dissection + ALLI (*n* = 1)	- ALLI o ALLI + PsA: SVPA + thrombectomy (*n* = 4) - PsA: SVPA (*n* = 1) - PsA + AVF: SVPA + venorraphy (*n* = 1) - PsA + bleeding: SVPA (*n* = 1) - CFA dissection + ALLI: SVPA + thrombectomy (*n* = 1) Most cases with PDS 7/0 suture	Follow-up: 9 mo (1.8 to 77.6) All children regained normal circulation, which was defined by means of palpable pedal pulses
Salvino [8] (2009)	Case report/1	1 m F/failed femoral vein cannulation	Right EIA and CFA/ALLI, thrombosis	Systemic heparinization first, after 96 h = embolectomy 2 Fr Fogarty catheter + 4 compartment fasciotomy	Reintervention for groin hematoma 24 h later Follow-up: 1 mo, with triphasic signals in the femoral artery by Doppler
Tasar [9] (2014)	Cases report/2	10 d M, patent ductus arteriosus	Right CFA/TCC/ALLI/artery transection	Embolectomy 2 Fr Fogarty catheter and GSV interposition	Follow-up: NS, only reported as a “long-term follow-up”, colored Doppler USG showed that graft was patent, and no ischemia was observed
		And 2 y F, patent ductus arteriosus and Pulmonary valve stenosis	Right CFA/TCC/ALLI, thrombosis/EIA occlusion secondary to Amplatzer occluder migration	Iliofemoral bypass with 8 mm PTFE graft	Follow-up: 24 mo, duplex-ultrasonography showed that bypass graft was patent and physical examination was normal
Andraska [3] (2017)	Retrospective cohort/ 81, 15 underwent surgery: 8 for ALLI	39 M, 35 F/17 mo (1 day to 17 years) Surgery patients: ALLI = 10.8 y (range, 7 y to 17y) ECMO (*n* = 4) IIDS (*n* = 2) AML (*n* = 1) Multiple (*n* = 1)	CFA/ALLI (74): - Hemodynamic monitoring (*n* = 52) - TCC or DCC (*n* = 12) - Cannulation for ECMO (*n* = 7),—IIDS (*n* = 2), ALLI, thrombosis in surgical patients: - Iliofemoral (*n* = 3) - FCA (*n* = 3) - NS (*n* = 2) - PsA(*n* = 1) - Arterial dissection + thrombosis (*n* = 1)	- 92% (n = 68) a received anticoagulant treatment (LMWH) - 10.8% (*n* = 8) received surgery: - SVPA (*n* = 2) -Thrombectomy + AISR (*n* = 1) - Thrombectomy + SVPA + fasciotomy (*n* = 1) - Bypass with PTFE graft (*n* = 1) - Thrombectomy + SVPA + fasciotomy + amputation (*n* = 1) -Bilateral BTK amputation (*n* = 1) - AVF and Psa ligation (*n* = 2)	Follow-up: 6 mo (range, 0 to 16) in surgical patients Complication (*n* = 4): - Chronic DVT (*n* = 1) - Graft stenosis (*n* = 1) - Amputation revision (*n* = 1) - Reoperation for wound dehiscence (*n* = 1) 88% of limb salvage
Beşir [10] (2017)	Retrospective cohort/17	11 F, 6 M/60.7 mo (SD 54.4)/ SeD (*n* = 7) TOF (*n* = 4) PDA (*n* = 2) AoCo (*n* = 2) NoCC (*n* = 2)	Right CFA (*n* = 11), left CFA (*n* = 3), left EIA (*n* = 1)/ -TCC or DCC (*n* = 15), - Surgical injury (*n* = 1) - External injury (*n* = 1) -ALLI, thrombosis (*n* = 10) - Hemorrhage (*n* = 5)	- AISR (*n* = 15) - E–E anastomosis (*n* = 1) - GSV graft interposition (*n* = 1) with 6/0 or 7/0 polypropylene suture - thrombectomy before repair (*n* = 16)	Follow-up: NS No mortality, no limb loss or infection
LoGiudice [11] (2017)	Retrospective cohort/1	1 day F/transposition of great arteries	Right CFA and EIA/DCC/bleeding and ALLI secondary to EIA's avulsion	Iliofemoral bypass with GSV 6 cm of length using 9/0 suture	Follow-up: 36 mo, leg perfused
Şişli [12] (2019)	Case report/1	11 mo F/PDA	Right CIA after TCC/ALLI secondary to arterial dissection	Laparotomy, and CIA to EIA bypass with PTFE	Follow-up: 5 mo No signs of limb ischemia and graft permeability
Author’s cases (2022)	Cases report/3	12 y F/AoCo	Right CFA occlusion secondary to EIA eversion after Sheath retrieval/TCC/ALLI	Right EIA to CFA 6 mm PTFE graft bypass	Follow-up: 6 mo, bypass patency, palpable pulses, no claudication Reoperation 4 d after surgery due to retroperitoneal hematoma; internal iliac artery was ligated
		2 years/21 trisomy and pneumonia	R CFA/ALLI/thrombosis after failed venipuncture	Right femoral thrombectomy with 2Fr Fogarty and AISR	Follow-up: 8 mo, no signs of recurrent ischemia, normal growth chart as it was expected
		9 y M/SeD	ALLI, thrombosis, secondary to DCC/Right EIA and CFA	Thrombectomy with 2Fr Fogarty and AISR with 7/0 polypropylene suture	Follow-up: 2 y No claudication

*M* male; *F* female; *mo* months; *y* years; *w* week; *d* days; *EIA* external iliac artery; *CIA* Common iliac artery; *CFA* common femoral artery; *SFA* superficial femoral artery; *TCC* Therapeutic Cardiac catheterization; *DCC* diagnostic cardiac catheterization; *BPG* blood pressure gradient between upper and lower extremities; *AISR* arterial interrupted suture repair; *IIDS* iatrogenic injury during surgery; *NS* not specified; *PTFE* polytetrafluoroethylene; *ALLI* acute lower limb ischemia; *PsA* Pseudoaneurysm; *AVF* arteriovenous fistulae; *SVPA* Saphenous vein patch angioplasty; *NoCC* noncardiac causes; *DSA* intraarterial digital subtraction angiography; *SeD* septal defect; *TOF* tetralogy of Fallot; *ARDS* acute respiratory distress syndrome; *AoCo* aortic coarctation; *PDA* patent ductus arteriosus. *ECMO* Extracorporeal membrane oxygenation; *LMWH* low molecular wight heparin, *DVT* deep vein thrombosis

Chronic complications were treated in 27 patients (16% of revised cases) with a mean age of 5.8 years (S.D. 1.8 years), 53% female, clinically determined by claudication or gait alterations (*n* = 14), and size discrepancy of the lower limbs during patient growth (*n* = 11), 2 cases was not specified the symptomatology. Surgical bypass was the commonest procedure to treat chronic complications. The most performed bypass was the iliofemoral with GSV (*n* = 20) and PTFE (*n* = 1), followed by femoro-femoral bypass (*n* = 5). The post-treatment outcomes were satisfactory, with 100% of limb salvage and a mean follow-up of 68 months (range from 1 month to 25.1 years). Outcomes were reported as graft patency, GSV graft dilatation (9 patients), ABI (ankle–brachial index) differences before and after bypass, and limb-length discrepancy diminution. Five children had limb-length discrepancy, which markedly improved after late revascularization. Finally, bypass complications were two graft stenosis and one reoperation due to graft occlusion. Table 2 resumes data on chronic limb ischemia secondary to iatrogenic trauma.

**Table 2:** Chronic complications

Author years	Type of study/Number of patients	Mean age/Gender/underlying condition	Anatomical site of injury/trauma mechanism/type of injury	Type of repair	Results and follow-up
Lin^2^ (2001)	Retrospective cohort (34 cases), 7 underwent surgery for CLI	4.8 y (Range, 1 w to 17.4 y)/ M = 4, F = 3/NS	CLI (*n* = 7) CFA/NS	- Ileofemoral bypass with GSV (*n* = 5), - Femoro-femoral bypass with GSV (1), or CFA SVPA (*n* = 1)	Follow-up: 40.1 mo (range of 1.9 to 5.1 y), all patients with palpable pedal pulses
Cardneau^13^ (2001)	Retrospective cohort of 12	7 y (range, 2 to 11 y)/M = 8, F = 4	CLI secondary to stenosis due to DCC or TCC: - Left iliofemoral (*n* = 4) - Right iliofemoral (*n* = 6) - Right aortofemoral (*n* = 1) - Right femoropopliteal (*n* = 2)	- Iliofemoral bypass with GSV (*n* = 10) Aortofemoral bypass with GSV (*n* = 1) Femoro femoral bypass with GSV (*n* = 1)	Follow-up: 9.4 y (range of 1.6 to 25.1 y) Mean dilatation of GSV of 35% (range 0 to 50%) Mean ABI of 0.97 post bypass vs. 0.7 preoperatively (*p* < 0.05) and diminution of LLD
Andraska^3^ (2017)	Retrospective cohort of 81, 7 underwent surgery for CLI	M = 3, F = 4/ 42 mo (range, 15 to 72)/NS	CLI secondary to stenosis due to DCC or TCC: - iliofemoral (*n* = 5) - CFA (*n* = 2)	Revascularization (*n* = 7): - Iliofemoral bypass with GSV (*n* = 5) - Iliofemoral bypass with PTFE (*n* = 1) - Femoral-SFA bypass with GSV(*n* = 1)	Follow-up: 50 mo (range, 1 to 118 mo) 2 graft stenosis, 1 reoperation due to graft occlusion 100% of limb salvage
Schwartz^15^ (2020)	Case report/1	8 year F/AoCo	CLI secondary to stenosis of R EIA and CFA after TCC at 28 weeks for aortic coarctation	SFA and profunda femoris artery bypass with GSV with 8/0 polypropylene suture	Follow-up: 6 y (72 mo), bypass patency. And normal growth chart as expected

*M* male; *F* female; *mo* months; *y* years; *w* week; *d* days; *EIA* external iliac artery; *CIA* Common iliac artery; *CFA* common femoral artery; *SFA* superficial femoral artery; *TCC* Therapeutic Cardiac catheterization; *DCC* diagnostic cardiac catheterization; *BPG* blood pressure gradient between upper and lower extremities; *AISR* arterial interrupted suture repair; *IIDS* iatrogenic injury during surgery; *NS* not specified; *PTFE* polytetrafluoroethylene; *ALLI* acute lower limb ischemia; *PsA* Pseudoaneurysm; *AVF* arteriovenous fistulae; *SVPA* Saphenous vein patch angioplasty; *NoCC* noncardiac causes; *DSA* intraarterial digital subtraction angiography; *SeD* septal defect; *TOF* tetralogy of Fallot; *ARDS* acute respiratory distress syndrome; *AoCo* aortic coarctation; *PDA* patent ductus arteriosus. *ECMO* Extracorporeal membrane oxygenation; *LMWH* low molecular wight heparin. *LLD* Limb length discrepancies

## Discussion

Along with the increase of endovascular therapies, vascular access complications are growing, and although more evidence about this topic would be expected, the number of reports has not increased over the years [14–16]. Pediatric cardiac catheterization, mainly when therapeutic procedures and large caliber devices are used, is related to a higher incidence of iatrogenic iliofemoral arterial injury, for example: during angioplasty, septal closure device or stent release, or even when the patient requires extracorporeal membrane oxygenation [17].

The literature shows that 2–4% of pediatric catheterizations may have some degree of injury. However, this proportion may increase up to 40% in presence of risk factors. Some authors propose that "the younger the age, the greater the risk of injury" due to increased vessel mobility and susceptibility to vasospasm, smaller size, and sharper angulations than older patients [1, 2, 11]. Vitellio et al. [16] and Kim et al. [18] mentioned that those patients under 1 year of age or with less than 8 kg of weight are even at a higher risk of injury (1.3% vs. 0.3% in children > 1 y *p* = 0.001). Lin et al. [2] identified statistically significant risk factors for arterial injury, including those younger than 3 years, three previous catheterizations, type of therapeutic intervention, and use of 6 Fr or larger endovascular devices.

Another possible factor contributing to vascular injury during femoral catheterization is the diameter of endovascular devices, because it is usually determined by the weight and age of the patient instead of the measured vessel lumen diameter. For example, > 6 Fr devices in patients younger than 1 year or < 10 kg is a risk factor for complications. Thus, device mismatch is a latent danger for patients facing obesity or malnutrition. Catheters that occupy > 50% of the arterial diameter or have < 1.9 mm of surrounding free space can cause vasospasm and possible arterial injury. In our case, another detrimental factor that should be considered is that hypoplasia of the abdominal aorta and its branches associated with AoCo [19].

Thus, Tadphale et al. suggest pre-catheterization routine diameter evaluation of the iliofemoral arteries by ultrasound (US) imaging in every pediatric patient. Key take away points are understanding the catheter size to fit into a sheath is based on sheath inner diameter (ID) but the impact on the choice of sheath size is based on the outer diameter (OD) of the sheath. The largest OD should never occlude the artery, since it increases the likelihood of an intimal injury and thrombosis. Thus, it one needs to know the size of the vessel lumen before choosing the equipment. For example, the ODs of a 7 Fr sheath is 2.5 to 3 mm, so, the arterial inner diameter must be > 3 mm. Regarding CFA diameter, if it is less than 3 mm, it could be a risk factor for loss of pulse after catheterization (OR 8.44, 95% Confidence interval 2.07–34.5, *p* < 0.001), irrespective of the patient size or age [20].

Also, when the percutaneous procedures are taking place, we suggest the performance of initial arteriography to thoroughly assess the puncture site and vessel diameter once the introducer has been placed and before placing any larger caliber devices. The preoperative instauration of a routine ultrasound-guided puncture would also considerably reduce the number of punctures and complications. Even in neonates, a 10 to 13 MHz transducer has been proven as an effective tool for femoral vessel evaluation and reduce puncture complications [20].

Conversely, this review found ALLI is the most common injury finding, secondary to thrombosis. Although up to 70–90% of cases could improve with anticoagulation alone, many patients will require other treatment modalities, such as systemic thrombolysis or surgery. Kayssi et al. [21] reported a cohort of 151 patients (84% < 1 year), of which 91% were due to iatrogenic injuries, 42% involved EIA, and 30% CFA. Six percent of patients received systemic thrombolysis when anticoagulation was insufficient; half did not respond to thrombolysis and required thrombectomy, fasciotomy, or amputation.

In 2006, Lazarides et al. [22] reported 23 children aged 13 years or younger with arterial extremity trauma (including other mechanisms than catheterization). They concluded that school-aged children (> 6 years) could safely undergo surgical repair, but neonates, infants, and preschool children are best treated non-operatively if they have an ischemic but nonthreatened extremity, concluding too if a distal Doppler signal was present, limb loss is rare. In the same study, patients treated non-operatively received systemic heparin or subcutaneous enoxaparin, the limb-length discrepancy was noted in only one patient at follow-up. They reported an 87% limb–salvage rate with this medical approach.

However, when ALLI are treated conservatively, strict long-term follow-up are needed to focus on symptoms, such as ABI detriment, claudication or gait alterations, and size discrepancy of the lower limbs during patient growth. Although children have a greater capacity to form collateral circulation, most of them could persist whit hypoperfusion of the affected limb. In 1983, Flanigan et al. [23] reported a 23% incidence of leg-length discrepancy after nonoperative treatment vs. a 9% incidence after surgical treatment as result of ischemia lasting more than 30 days. A difference of 2 cm or more between both legs could be detrimental.

When ALLI requires surgical treatment, the most performed procedures were embolectomy through a femoral approach. Most authors used a 2Fr Fogarty catheter for thrombectomy and accomplished the arterial repair employing arteriorrhaphy or GSV patch angioplasty (SVPA). Most reports agree that arteriorrhaphy execution with single interrupted sutures is the preferred approach using polypropylene or polydioxanone sutures ranging from 6/0, 9/0 to 11/0 (in arteries < 1.5 mm diameter), being 9/0 the most used [2, 15, 24, 25]. LaQuaglia et al. [26] mentioned that an arterial interrupted suture repair in vessels larger than 1 mm in diameter using microsurgical techniques (microscope with X8 magnification and 9/0 to 11/0 sutures) has a 90% patency rate at long-term follow-up.

The great saphenous vein (GSV) has been used in transposition, bypass, and as a patch for angioplasty, as it has growth potential. However, D’oria and Cols. [25] reported a case in which there was a 1:3 mismatch between the GSV and the CFA, and they opted to use the femoral vein as a graft. PTFE has been reported as a graft in children from 11 months (4 mm graft) to 12 years, an 8 mm graft in a 2-year-old boy for iliac segment reconstruction, and a 6 mm graft in a 5-month-old patient who underwent a fem-pop bypass as well. However, they suggested that graft often leads to less satisfactory outcomes in children younger than 2 years [2, 9].

In a cohort of 33 patients who underwent vascular repair between 2002 and 2017, Kampf et al. [27] reported performing 15 bypass procedures in pediatric patients, of which six were secondary to trauma in the iliofemoral sector (not clear if trauma was iatrogenic). The GSV has the potential for stretching and thickening, which may be consistent with the axial growth of the pediatric patient. Prosthetic grafts do not have that quality. In our case 1, it was used, because GSV was inappropriate and because the patient had already presented her greatest axial growth peaks. Growth after menarche is variable, between 4 and 12 cm, although most girls grow only about 6–7 cm and most of it in the first and second year after the first period [14].

On the other hand, in a contemporary series from the National trauma databank, 62% of pediatric trauma cases (both civil and iatrogenic) were treated surgically, 30.7% conservatively, and 7.3% endovascularly. The latter has been increasing (5% in 8 years), being angioembolization of internal iliac injury and thoracic aortic endograft placement the two most common endovascular procedures (33.4% and 22.9%, respectively) in patients with a mean age of 14 years [28]. In the case we presented, endovascular treatment was not feasible, because the EIA eversion prominently obstructed the CFA lumen and had to be surgically removed. On the other hand, the GSV did not have a sufficient diameter to be used as a graft due to vessel hypoplasia. Since the patient had iliofemoral arterial hypoplasia and had also completed 2/3 of her body growth, we decided to use a 6 mm graft, adjusted to the dimensions of her vessels, but we cannot rule out possible future reinterventions.

Limitations to this study include the small number of studies published on this subject in the pediatric population and the impossibility of further delimiting the anatomical vascular region without affecting the reported outcomes, since most reviewed articles do not specify the correlation of the anatomical site with the outcome. Finally, the indications of cardiac catheterization, device diameter, and the number of arterial punctures were not specified in most of the included patients, making it difficult to make direct comparisons between patients.

## Conclusion

Iatrogenic iliofemoral artery injury in the pediatric population frequently occurs after percutaneous cardiac catheterization. Therefore, further reports about its prevention, treatment, and long-term follow-up are needed.

## Data Availability

No applicable, because no database was constructed, and all data were obtained of patient’s charts and the review information is available in the articles enlisted in references. Nonetheless, signed informed consent are available, if necessary, with the corresponding author.
